# The impact of self-reported burnout and work-related quality of life on nurses' intention to leave the profession during the COVID-19 pandemic: A cross-sectional study

**DOI:** 10.3934/publichealth.2024056

**Published:** 2024-11-07

**Authors:** Susan McGrory, John Mallett, Justin MacLochlainn, Jill Manthorpe, Jermaine Ravalier, Heike Schroder, Denise Currie, Patricia Nicholl, Rachel Naylor, Paula McFadden

**Affiliations:** 1 School of Nursing and Paramedic Science, Magee Campus, Ulster University, Derry, BT48 7JL, UK; 2 School of Psychology, Coleraine Campus, Ulster University, Coleraine BT52 1SA, UK; 3 School of Applied Social Policy Sciences, Ulster University, Derry BT48 7JL, UK; 4 NIHR Policy Research Unit in Health and Social Care Workforce, King's College London, Strand, London WC2B 6LE, UK; 5 School of Health and Social Care Professions, Buckinghamshire New University, High Wycombe, HP11 2JZ, UK; 6 Queen's Business School, Queen's University Belfast, Belfast BT9 5EE, UK; 7 School of Social Sciences, Education and Social Work, Queen's University Belfast, Belfast BT7 1HL, UK

**Keywords:** nursing, burnout, quality of working life, retention, COVID-19, UK

## Abstract

The challenges of maintaining an effective and sustainable healthcare workforce include the recruitment and retention of skilled nurses. COVID-19 exacerbated these challenges, but they persist beyond the pandemic. We explored the impact of work-related quality of life and burnout on reported intentions to leave a variety of healthcare professions including nursing. We collected data at five time-points from November 2020 to February 2023 via an online survey. The validated measures used included the Copenhagen Burnout Inventory and Work-Related Quality of Life (WRQoL) scale; with subscales for Job-Career Satisfaction, General Wellbeing, Control at work, Stress at work, Working conditions, and Home-work interface. Our findings showed that 47.6% of nursing respondents (*n* = 1780) had considered changing their profession throughout the study period, with the 30–39-year age group most likely to express intentions to leave. Regression analysis reveale that for WRQoL, lower general wellbeing and job-career satisfaction scores predicted intentions to leave when controlling for demographic variables (*p* < 0.001). When burnout was added to the regression model, both work-related and client-related burnout were predictive of intentions to leave (*p* < 0.001). These findings highlighted that significant numbers of nurses considered leaving their profession during and shortly after the pandemic and the need for interventions to improve nurses' wellbeing and reduce burnout to improve their retention.

## 1. Introduction

As the largest group of healthcare workers, the recruitment and retention of nurses is of major concern across the world with a predicted global shortage of nurses of up to 10 million by 2023 [1],[2]. While the COVID-19 pandemic was considered to affect the intention to leave the nursing profession [3],[4], the situation was recognized as problematic well before the pandemic [1]. In the UK, 2022–2023 saw the highest number of new registrants on the Nursing & Midwifery Council (NMC) register, many of whom trained outside the UK. While there was a slight decrease in those who left the profession (1.4%) compared to the previous year, over half of those leaving the register (52.1%) left earlier than planned, with a quarter leaving much earlier than planned [5]. In the UK, while nurses and health visitors (generally nurses specializing in community-based family health) represent 26% of full-time equivalent (FTE) posts, they are overrepresented in the total National Health Service (NHS) vacancies at 35% [6]. This highlights the need to address recruitment as well as retention within this workforce.

Intention to leave is important as it has been identified as a predictor of leaving behavior among nurses [7],[8] and may impact on turnover which, in turn, may have a detrimental effect on patient care and outcomes [9]. However, literature does not always differentiate clearly between intention to leave current post and intention to leave the profession of nursing [10]. For example, some studies focus on nurses' intention to leave their current post [11]–[13], while others address the intention to leave the profession [14]–[16].

Intention to leave numbers vary widely across countries, with a European study in 2013 reporting rates from 5%–17% in European-based nurses [17]. Reported rates elsewhere also vary, ranging from 2% in the United States (US) [18], 10% in Thailand [19], 22.3% in Canada [20] and 33.1% in Italy [14] to 24.6% in Brazil [15]. A more recent study across six European countries reported that 33% of nurses expressed an intention to leave [21].

Early studies of burnout, described it as a depleted state of energy associated with an individual's experience of human services' work, which is characterized by emotional exhaustion, depersonalization, and reduced feelings of accomplishment [22]. More recently, Kristenson et al. (2005) suggest that core components of burnout are fatigue and exhaustion and how these are attributed to the specific domains of personal life, working life and client related work [23]. High rates of nursing burnout have been reported both prior to the COVID-19 pandemic [24] as well as during the pandemic [25],[26]. Burnout has been identified as a strong predictor of intention to leave [19],[27]–[30] and in one international study was the one factor found to consistently predict intention to leave the profession across all 10 countries studied [17].

Age is also found to be a predicting factor of intention to leave the profession in a range of studies [3],[31],[32]. The leaving behavior of newly qualified nurses was explored by Zhang et al. (2017), with intentions to leave largely predicted by levels of occupational stress and a lack of professional identity [33]. Newly qualified staff were identified as an at-risk group that are particularly important to retain [34],[35]. For example, Mulud et al. (2022) found that 12.4% of newly graduated nurses intended to leave their profession, with a significant positive correlation between intentions to leave and levels of stress [36]. This highlights the necessity for interventions to address the needs of newly qualified staff and younger staff to sustain the future workforce.

A range of factors related to working life and experience has also been linked to the intention to leave. Quality of working life refers to a person's satisfaction with their working life that is impacted by perceptions and feelings [37]. It is further suggested that quality of working life is affected by a range of direct and indirect factors that impact an individual's experience of work including their wellbeing, working conditions, as well as perception of control and stress at work [38]. Job satisfaction, influenced by factors such as work-life balance [16],[28], working conditions [39] and staffing and conditions [40],[41] may also influence nurses' intention to leave. Some studies published during the pandemic also suggest that the pandemic directly impacted intentions to leave [42]. The factors that affect turnover are, therefore, complex [43] and it is vital to develop a detailed understanding of these to develop interventions to improve retention.

In view of this complexity, we aimed to explore a range of factors that may influence intention to leave the nursing profession including demographic characteristics, burnout, and work-related quality of life. As the study was conducted at five time points during the pandemic, it provided the opportunity to explore changes in intention to leave over time. We hypothesized that higher rates of burnout and lower quality of working life would increase the likelihood of nurses reporting intentions to leave the profession.

## 2. Materials and methods

### 2.1. Design

This study formed part of a larger multiphase research program, which examined quality of working life and coping among various UK health and social care workers at different stages of the COVID-19 pandemic. The overall study adopted a cross-sectional design with data collected at six time points commencing in May 2020. The data collection phases were Phase 1: May–July 2020; Phase 2: November 2020 to February 2021; Phase 3: May to July 2021, Phase 4: November 2021 to February 2022; Phase 5: May–July 2022; and Phase 6: November 2022-February 2023.

Data were collected via an online survey which included the use of validated measures of work-related quality of life and burnout as well as demographic questions that included gender, age, country of work (Northern Ireland (NI), Scotland, England, Wales), work setting, area of practice, and from Phase 2, participants were asked about their intentions to leave their profession. A select number of open-ended questions afforded participants the opportunity to elaborate on their experiences. The data analyzed in this paper derive from phases 2–6 quantitative data and includes only registered nurses.

### 2.2. Measures

#### 2.2.1. Quality of working life: Work-Related Quality of Life scale (WRQoL) [44]

The Work-Related Quality of Life (WRQoL) scale was used to explore quality of working life [44]. The scale contains 23 items which assess six domains of working life. These include Career Job Satisfaction (six items), General Wellbeing (six items), Control at work (three items), Stress at work (two items), Working conditions (three items), and Home-work interface (three items). One additional question on overall wellbeing is not included in the final score. Respondents rate items through a 5-point Likert scale from 1 ‘Strongly Disagree’ to 5, ‘Strongly Agree’. Total scores for each subscale were computed by summing the relevant items, with better quality of working life indicated by higher scores. Health service norms were reported by Easton & van Laar (2018) [44], and scores can be divided into lower, average, and higher quality of working life with the cut-off points identified in Table 1. Individual scores for each domain can be calculated, with the domain ‘stress at work’ being reverse scored and for this category, lower scores indicate higher levels of stress at work [44]. The WRQoL sub-scales demonstrated good internal consistency previously in the present study with Cronbach's alpha coefficients ranging from (*α* = 0.77–0.87).

**Table 1.** WRQoL cut-off scores [44].

Quality of working life	WRQoL Domain						WRQoL score overall
	Job career satisfaction	Stress at work	General wellbeing	Control at work	Working conditions	Home-work interface	
Lower	6–19	2–4	6–20	3–8	3–9	3–9	23–71
Average	20–22	5	21–23	9–10	10–11	10–11	72–82
Higher	23–30	6–10	24–30	11–15	12–15	12–15	83–115

#### 2.2.2. Burnout: Copenhagen Burnout Inventory [23]

The 19-item Copenhagen Burnout Inventory [23] was used to measure three areas of burnout including work-related burnout (seven items), personal burnout (six items), and client-related burnout (six items). The mean score for each area of burnout is calculated as a score from 0–100, with higher scores indicating a higher level of burnout. The scores were categorized into low, medium, and high levels of burnout using previously cited cut-off points [45] (Table 2). In this study, internal consistency was good with Cronbach alpha for personal burnout, *α* = 0.90, work-related burnout *α* = 0.90 and client-related burnout *α* = 0.88.

**Table 2.** Categorization of burnout scores.

Level of burnout	Cut off scores
Low	0–49
Moderate	50–74
High	75–99
Severe	100

### 2.3. Data analysis

Data were prepared for analysis, separating extraneous data to include only nurses from Phase 2 to Phase 6 (*n* = 1740). SPSS version 28 was used to generate descriptive statistics. Hierarchical logistic regression was conducted to compare various predictors of intention to leave the profession.

#### 2.3.1. Treatment of missing data

The percentage of missing data on each of the categorical demographic variables was in the range (0%–1.6%) but was notably higher for the WRQoL and Burnout subscale total score variables. The missingness percentages for the WRQoL subscales were in the range (10.5%–11.7%) and the Copenhagen Burnout sub-scales were in the range (12.1%–19.2%). The percentage of missing data on the outcome variable (Intention to Leave) was 7.9%.

This effectively reduced the initial sample size of 1740 to 1389 due to the listwise (casewise) deletion procedure in logistic regression. Little's Missing Completely at Random (MCAR) test indicated that the missingness was indeed completely random χ^*2*^(90) = 95.59, *p* = 0.324 implying that using listwise deletion is unlikely to have biased the results in the logistic regression models.

### 2.4. Sample

The sample was recruited through professional organizations, unions, and employers (Northern Ireland only) as well as social media platforms, including Facebook and Twitter (now X) and was therefore opportunistic in nature.

### 2.5. Ethics

Ethical approval was granted by the Filter Committee School of Nursing and Paramedic Science, Ulster University (Ref No. 2020/5/3.1) Trust governance approval for Northern Ireland only from Phase 2 allowed the survey to be shared directly with Health and Social Care staff. Permissions were granted by authors of the original scales used for measurement within the survey. A participant information sheet was provided on accessing the survey link, addressing anonymity and consent, and containing contact details for the research team. At the end of the survey, relevant support information for respondents who may have been experiencing distress was provided.

## 3. Results

### 3.1. Sample characteristics

Table 3 presents the overall sample characteristics of nurses only across the phases of the study. The sample was predominantly female over the five phases at 89.9%, with males comprising 9.6% of the respondents. The major areas of practice were Adults (38.7%), Mental Health (14.5%), and Older People (18.3%), and most nurses reported working in a hospital (45.9%) or community (28%) setting. Of the sample, 45% of nurses were aged 50 years and over with 55% aged 49 years or younger. The number of respondents across the individual phases ranged from 218 to 566.

**Figure 1.**
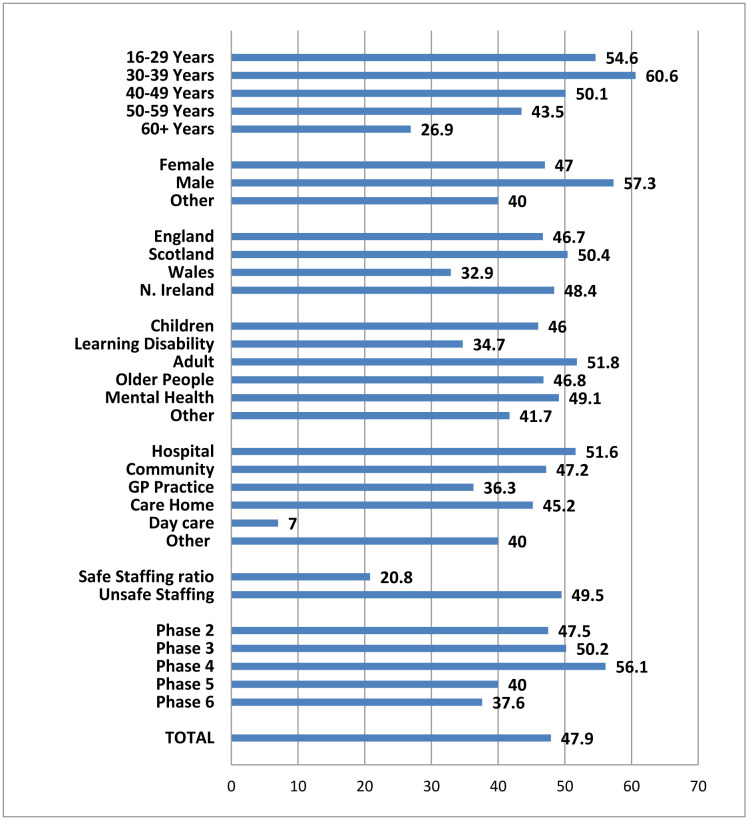
Percentages of the sample (*N* = 1602/1938) reporting intention to leave by demographic characteristics, region, service area, service location, and phase of research.

Descriptive statistics for the outcome variables are presented in Tables 4–5/Figure 1. Overall, nearly half, 47.9%, of nurses reported their intentions to leave the profession. Figure 1 identifies the percentages of nurses who reported intentions to leave by age, gender, country, area of practice, setting and phase. In Phase 6, respondents were asked about their perception of safe staffing in their setting and the percentage identifying intentions to leave in relation to safe/unsafe staffing is also presented in Figure 1. As this question was asked only in Phase 6, it was not possible to include this factor in the final regression model. However, in Phase 6, those who felt they were not operating under safe staffing conditions were significantly more likely to express an intention to leave *c^2^* (1) =14.95, *p* < 0.001.

Table 4 presents the means domain scores for WRQoL scores and the totals across the phases. Throughout the study period, scores indicated for the domains of stress at work (reversed scored), and general wellbeing, remained low. Working conditions scores in Phase 6 were also low. All other scores were average, although throughout remained at the lowest score possible to be regarded as average according to health service norms [44].

Table 5 reports the means of the components of the Copenhagen Burnout Inventory for each of the phases. Burnout scores reveal that both work-related and personal burnout scores remained at moderate levels on average, whereas client-related burnout scores remained low. ANOVA tests revealed that work-related burnout and personal burnout scores increased significantly between phases 3 and 5 (*p* = 0.007 and *p* = 0.006, respectively).

**Table 3.** Sociodemographic details for nurses, phases 2–6 (*n* = 1740).

Variable	Phase 2, *n* (%) (November 2020–February 2021)	Phase 3, *n* (%) (May–July 2021)	Phase 4, *n* (%) (November 2021–February 2022)	Phase 5, *n* (%) (May–July 2022)	Phase 6, *n* (%) (November 2022– February 2023)
Gender					
Female	325 (90)	508 (89.8)	325 (90)	208 (88.9)	199 (91.3)
Male	35 (9.7)	56 (9.9)	34 (9.4)	24 (10.3)	18 (8.3)
Other	1 (0.3)	2 (0.4)	2 (0.6)	2 (0.9)	1 (0.5)
Age (years)					
18–29	36 (10)	61 (10.8)	26 (7.2)	23 (9.8)	23 (14)
30–39	58 (16.1)	106 (18.7)	70 (21.2)	49 (14.8)	47 (14.2)
40–49	108 (29.9)	134 (23.7)	94 (26)	61 (13.3)	61 (13.3)
50–59	118 (18.7)	220 (34.9)	141 (22.3)	81 (12.8)	71 (11.3)
60+	41 (11.4)	45 (8.0)	30 (8.3)	20 (8.5)	16 (11.8)
Country of work					
England	81 (22.4)	69 (12.2)	62 (17.2)	73 (31.2)	25 (11.5)
Scotland	16 (4.4)	276 48.8)	137 (38.0)	7 (3.0)	17 (7.8)
N. Ireland	214 (59.3)	206 (36.4)	152 (42.1)	142 (60.7)	174 (79.8)
Wales	50 (13.9)	15 (2.7)	10 (2.8)	12 (5.1)	2 (0.9)
Place of work					
Hospital	135 (37.4)	301 (53.3)	176 (48.8)	81 (34.6)	106 (48.6)
Community	116 (32.1)	142 (25.1)	96 (26.6)	76 (32.5)	57 (26.1)
GP based	17 (4.7)	19 (3.4)	13 (3.6)	23 (9.8)	24 (11.0)
Day care	2 (0.6)	5 (0.9)	1 (0.3)	2 (0.9)	2 (0.9)
Care home	43 (11.9)	28 (5.0)	14 (3.9)	22 (9.4)	5 (2.3)
Other	48 (13.3)	70 (12.4)	61 (16.9)	30 (12.8)	24 (11.0)
Main area of practice					
Children	44 (12.4)	66 (11.9)	45 12.6)	19 (8.3)	28 (12.8)
Adults	134 (37.9)	281 (50.5)	99 (27.8)	70 (30.7)	79 (36.2)
Learning Disability	17 (4.8)	19 (3.4)	8 (2.2)	6 (2.6)	2 (0.9)
Older people	62 (17.5)	71 (12.8)	77 (21.6)	68 (29.8)	35 (16.1)
Mental health	71 (20.1)	68 (12.2)	53 (14.9)	29 (12.7)	27 (12.4)
Other	26 (7.3)	51 (9.2)	74 (20.8)	36 (15.8)	47 (21.6)
Considering leaving profession		
Yes	150 (47.5)	259 (50.2)	192 (56.1)	84 (40)	82 (37.6)
No	166 (52.5)	257 (49.8)	150 (43.9)	126 (60)	136 (62.4)

**Table 4.** Mean scores for WRQoL across phases 2 to 6.

HSC study phase		WRQOL: Job career satisfaction	WRQOL: Stress at work	WRQOL: Working conditions	WRQOL: Control at work	WRQOL: General wellbeing	WRQOL: Home-work interface	WRQOL: Total score
Phase 2	*Mean*	20.78250	4.12620	9.84420	9.42020	18.77520	9.78390	72.82570
	*N*	308	309	308	307	307	310	304
	*SD*	4.60506	1.69422	2.72072	2.96207	4.61933	2.96938	15.10729
Phase 3	*Mean*	20.41380	4.66800	10.07100	9.40160	19.17650	9.98000	73.75460
	*N*	493	494	493	493	493	499	489
	*SD*	4.86016	1.96911	2.53051	2.90447	4.80126	2.95746	15.74076
Phase 4	*Mean*	20.69490	4.30720	9.92150	9.50760	19.34040	9.90360	73.66260
	*N*	331	332	331	331	332	332	329
	*SD*	4.73932	1.81689	2.79391	3.03252	4.68737	3.04990	15.85486
Phase 5	*Mean*	20.21940	4.27550	9.58670	9.61030	19.30260	9.46230	72.45130
	*N*	196	196	196	195	195	199	195
	*SD*	4.89247	1.91769	2.77306	3.15443	4.91219	3.05461	17.15248
Phase 6	*Mean*	20.75710	4.22380	9.40000	9.26670	19.15240	9.92170	72.73810
	*N*	210	210	210	210	210	217	210
	*SD*	4.75657	1.92749	3.13828	3.16157	4.81481	3.03351	17.37713
Total	*Mean*	20.57020	4.37120	9.84010	9.43620	19.14440	9.85040	73.24360
	*N*	1538	1541	1538	1536	1537	1557	1527
	*SD*	4.77216	1.88150	2.75178	3.00962	4.75506	3.00344	16.05037

**Table 5.** Mean CBI scores across phases.

HSC study phase		Client-related burnout	Work-related burnout	Personal burnout
Phase 2	*Mean*	25.84810	59.18030	62.10770
	*N*	283	305	308
	*Std. Deviation*	20.17280	20.75493	19.60734
Phase 3	*Mean*	25.47000	55.61540	59.25510
	*N*	461	484	490
	*Std. Deviation*	22.06686	22.42723	20.65120
Phase 4	*Mean*	27.63010	59.06370	61.31310
	*N*	301	326	330
	*Std. Deviation*	22.44859	21.69204	21.04781
Phase 5	*Mean*	27.78550	61.04570	65.14960
	*N*	181	194	195
	*Std. Deviation*	21.15974	21.89586	19.92242
Phase 6	*Mean*	28.72690	61.09230	63.46620
	*N*	180	200	207
	*Std. Deviation*	20.27175	22.91016	20.26920
Total	*Mean*	26.72360	58.50490	61.59420
	*N*	1406	1509	1530
	*Std. Deviation*	21.44597	22.01207	20.45799

Pearson zero-order correlations among the sub-scales of the Copenhagen Burnout Inventory (Table 6) showed a strong positive correlation between Personal and Work-related Burnout scores *r*(1507) = 0.78, *p* < 0.001.

Likewise, an examination of the correlations among the WRQoL subscales indicated that Job-Career Satisfaction was highly positively correlated with both Control at Work *r*(1685) = 0.73, *p* < 0.001 and Working Conditions *r*(1687) = 0.73, *p* < 0.001. Control at Work was likewise moderately positively correlated with Working Conditions *r*(1686) = 0.65, *p* < 0.001 and Home Work Interface scores *r*(1688) = 0.65, *p* < 0.001.

The strongest negative correlations were evident between scores on the General Wellbeing subscale of the WRQoL and both Personal Burnout *r*(1523) = −0.66, *p* < 0.001 and Work-related Burnout scores *r*(1502) = −0.64, *p* < 0.001. Finally, the Stress and Work WRQoL scores were negatively correlated with Work-related Burnout *r*(1506) = −0.60, *p* < 0.00.

**Table 6.** Pearson zero-order correlations between the subscales of the Work-Related Quality of Life Scale and the Copenhagen Burnout Inventory (Pairwise *N* = 1400–1692).

Project	1	2	3	4	5	6	7	8
1. Job-career satisfaction	-							
2. Stress at work	0.234	-						
3. Working conditions	0.732	0.354	-					
4. Control at work	0.760	0.207	0.647	-				
5. General wellbeing	0.598	0.434	0.595	0.499	-			
6. Home-work interface	0.670	0.309	0.649	0.587	0.537	-		
7. Personal burnout	−0.384	−0.490	−0.419	−0.332	−0.661	−0.422	-	
8. Work-related burnout	−0.495	−0.603	−0.588	−0.423	−0.643	−0.546	0.778	-
9. Client-related burnout	−0.277	−0.260	0.304	−0.231	−0.335	−0.248	0.321	0.427

Note: all correlations are statistically significant at *p* < 0.001 (two-tailed). Largest correlations are bolded.

To better understand any conceptual overlap between the six dimensions of the WRQoL and three CBI sub-scales, a principal component analysis was performed with ProMax rotation using the values from the correlation matrix shown in Table 6 as input. Results are summarized in Table 7 and indicated a correlated two-component solution based on the widely used Guttman-Kaiser criteria [46]–[48].

The first component loaded strongly on the WRQoL subscales of Job-career Satisfaction, Working Conditions, Control at Work and Home-Work Interface with standardized loadings in the high range (λ = 0.77–0.96) but lower on General Wellbeing (λ = −0.39) and Stress at Work (λ = −0.22). The second component indicated high loadings for the CBI subscales of Personal Burnout (λ = −0.86), Work-related Burnout (l = −0.83) and Client-related Burnout λ = −0.55) and included a strong loading from the WRQoL Stress at Work subscale (λ = −0.89) and a moderate loading for General Wellbeing (λ = −0.53).

**Table 7.** Pattern matrix of standardized rotated loadings following principal components analysis with promax rotation.

Sub-scales	Component 1	Component 2

	*‘Work-related quality of life’*	*‘Burnout’*
Job-career satisfaction	0.964	−0.088
Stress at work	−0.216	0.892
Working conditions	0.774	0.138
Control at work	0.960	−0.172
General wellbeing	0.388	0.533
Home-work interface	0.765	0.092
Personal burnout	0.005	−0.860
Work-related burnout	−0.132	−0.834
Client-related burnout	−0.006	−0.547
Eigenvalue (% Variance)	4.89 (54.32%)	1.30 (14.42%)

### 3.2. Logistic regression

Hierarchical logistic regression modeling was employed to compare incrementally complex sets of predictors, starting with demographic covariates (age group and gender in Model 1), and adding country in Model 2 (England, Scotland, Wales, Northern Ireland). Model 3 then included practice setting (hospital, community, General Practitioner (GP), care home, day-care, and others) and type of service area (children, learning disability, adult, older people, mental health, other), and the phases of data collection were then added to Model 4 (phases 2–6). Model 5 included all the WRQoL subscales (Job-career Satisfaction, Stress at Work, Working Conditions, Control at Work and General Wellbeing) and the final model 6 included the three Copenhagen Burnout subscales (Personal, Job-related and Client-related Burnout). The order in which sets of predictors entered the regression model was determined to assess the unique predictive relationships of the demographic, regional and service type variables in the first instance and to subsequently model the unique additive effects of each of the WRQoL and Burnout subscales using these initial variables as controls. The regression results are summarized in Table 8.

- In Model 1, Table 8 shows that all age groups were more likely than the 60+ age group to report intention to leave with odds ratios (*OR*) in the range 2.28 to 4.02 (Cox & Snell *R^2^* = 0.04, Nagelkerke *R^2^* = 0.06). The younger age groups aged 16–29 years and 30–39 years reported the largest likelihoods (*OR* = 4.02, 95% *CI* = 2.30–7.04 and *OR* = 4.53, 95% *CI* = 2.81–7.31 respectively). The older groups aged 40–49 years and 50–59 years were also more likely than the 60+ age group to report an intention to leave (*OR* = 2.99, 95% *CI* = 1.90–4.69.04 and *OR* = 2.28, 95% *CI* = 1.47–3.53 respectively). Men were also more likely to report an intention to leave (*OR* = 1.68, 95% *CI* = 1.15–2.45).
- In Model 2, no significant differences were evident between countries (Cox & Snell *R^2^* = 0.05, Nagelkerke *R^2^* = 0.06), but the pattern of results for age group and gender remained, with younger age groups (*OR* range = 2.25–4.42) and men (*OR*= 1.70, 95% *CI* = 1.16–2.48) reporting higher likelihood values.
- Model 3 indicated that those working in GP (Family Physician) practices were less likely to report an intention to leave compared to those working in hospitals (*OR* = 0.524, 95% *CI* = 0.32–0.87). In addition, adjusting for work setting and service area resulted in a small country difference with a lower intention to leave probability reported in Wales compared to England (*OR* = 0.50, 95% *CI* = 0.26–0.93). The pattern of results for age group and gender also remained, with younger age groups (*OR* range = 2.28–4.23) and men (*OR* = 1.73, 95% *CI* = 1.17–2.55) reporting higher likelihood values (Cox & Snell *R^2^* = 0.06, Nagelkerke *R^2^* = 0.08).
- Model 4 included time phases 2–6 as dummy covariates, with Phase 6 as the reference category (Cox & Snell *R^2^* = 0.07, Nagelkerke *R^2^* = 0.10). Intention to leave was more likely in Phase 2 (*OR* = 1.85, 95% *CI* = 1.10–2.49), Phase 3 (*OR* = 1.71, 95% *CI* = 1.16–2.54) and Phase 4 (*OR* = 2.31, 95% *CI* = 1.54–3.47). No significant difference emerged between Phases 5 and 6 (*p* > 0.05). In this model, the age and gender differences remained significant with younger age groups (*OR* range = 2.29–4.44) and males (*OR* = 1.71, 95% *CI* = 1.15–2.52) reporting higher likelihood values.
- Model 5 included all six of the WRQoL subscales (Cox & Snell *R^2^* = 0.07, Nagelkerke *R^2^* = 0. 10). Intention to leave likelihood was uniquely linked to lower Career Satisfaction (*OR* = 0.90, 95% *CI* = 0.86–0.94), higher Stress at Work (*OR* = 0.84, 95% *CI*= 0.77–0.90) and lower Wellbeing scores (*OR* = 0.92, 95% *CI* = 0.88–0.95). The phase differences remained significant in this model with a greater likelihood of intention to leave at Phase 2 (*OR* = 1.75, 95% *CI* = 1.11–2.78), Phase 3 (*OR* = 2.45, 95% *CI* = 1.56–3.85), and Phase 4 (*OR* = 3.23, 95% *CI* = 2.03–5.13). In this model, there were no significant differences between countries, work setting or service area (*p* > 0.05), but the age and gender differences remained consistently significant with younger age groups (*OR* range = 1.86–3.31) and men (*OR* = 1.83, 95% *CI* = 1.17–2.87) reporting higher likelihood values.
- Model 6 (see Table 7) included all three of the Copenhagen Burnout subscales (Cox & Snell *R^2^* = 0.27, Nagelkerke *R^2^* = 0. 36). Higher scores on Work-Related Burnout (*OR* = 1.83, 95% *CI* = 1.17–2.87) and Client-related Burnout (*OR* = 1.83, 95% *CI* = 1.17–2.87) were associated with greater intention to leave probabilities. Consistent with model 5, both lower Career Satisfaction (*OR* = 0.90, 95% *CI* = 0.86–0.95) and lower Wellbeing scores (*OR* = 0.95, 95% *CI* = 0.91–0.99) were linked to an increased likelihood of reporting intention to leave, but the Stress at Work was non-significant (*p* > 0.05), after adjusting for Burnout scores. There were no significant differences between countries, work setting or service area (*p* > 0.05), but the age and gender differences remained consistently significant with younger age groups (*OR* range = 1.71–2.69) and men (*OR* = 1.74, 95% *CI* = 1.17–2.87) reporting higher likelihood values.

**Table 8.** Binary logistic regression results (Model 6) for intention to leave (0 = No, 1 = Yes) on demographic variables, service area, work setting, time phase, and the subscales of the WRQoL Scale and the Copenhagen Burnout Inventory (Casewise *N* = 1389).

Covariates	*B*	*S.E*.	Wald	*df*	*p*	Odds Ratio (*OR*)	95% *CI* for *OR*	
							Lower	Upper
*Age group (ref. 60–69 years)*							
16–29	0.801	0.346	5.366	1	**0.021**	2.228	1.131	4.388
30–39	0.989	0.288	11.838	1	**<0.001**	2.690	1.531	4.725
40–49	0.687	0.273	6.359	1	**0.012**	1.988	1.165	3.392
50–59	0.539	0.262	4.235	1	**0.040**	1.714	1.026	2.864
*Gender (Male)*	0.555	0.237	5.476	1	**0.019**	1.742	1.094	2.774
*Country (ref. England)*								
Scotland	−0.267	0.211	1.595	1	0.207	0.766	0.506	1.159
Wales	−0.535	0.379	1.995	1	0.158	0.586	0.279	1.230
Northern Ireland	0.035	0.182	0.037	1	0.848	1.035	0.725	1.479
*Practice area (ref. Children)*							
Learning disability	−0.435	0.415	1.099	1	0.294	0.647	0.287	1.459
Adult	0.005	0.220	0.001	1	0.981	1.005	0.653	1.548
Older people	−0.135	0.248	0.295	1	0.587	0.874	0.538	1.420
Mental health	−0.377	0.251	2.250	1	0.134	0.686	0.419	1.122
Other (reference)	−0.191	0.252	0.575	1	0.448	0.826	0.504	1.353
*Work setting (ref. hospital)*							
Community	0.094	0.169	0.307	1	0.580	1.098	0.788	1.530
GP practice	−0.367	0.306	1.443	1	0.230	0.693	0.381	1.261
Care home	0.292	0.333	0.770	1	0.380	1.339	0.697	2.571
Day care	−1.326	1.125	1.390	1	0.238	0.265	0.029	2.407
Other	−0.199	0.207	0.927	1	0.336	0.819	0.546	1.229
*Phase (ref. Phase 6)*								
Phase 2	0.684	0.242	8.074	1	**0.004**	1.983	1.237	3.179
Phase 3	0.973	0.237	16.782	1	**<0.001**	2.645	1.661	4.213
Phase 4	1.181	0.242	23.881	1	**<0.001**	3.258	2.029	5.232
Phase 5	0.175	0.261	0.453	1	0.501	1.192	0.715	1.986
*Work-Related Quality of Life*							
Job/career satisfaction	−0.102	0.025	17.020	1	**<0.001**	0.903	0.860	0.948
Stress at work	−0.074	0.044	2.833	1	0.092	0.928	0.852	1.012
Working conditions	−0.007	0.038	0.034	1	0.853	0.993	0.921	1.071
Control at work	0.018	0.033	0.274	1	0.601	1.018	0.953	1.087
General wellbeing	−0.049	0.044	5.075	1	**0.024**	0.952	0.913	0.994
Home-work interface	−0.001	0.038	0.001	1	0.985	0.999	0.941	1.062
*Burnout*								
Personal burnout	−0.002	0.005	0.172	1	0.678	0.998	0.987	1.008
Work burnout	0.022	0.006	14.466	1	**<0.001**	1.023	1.011	1.035
Client burnout	0.019	0.003	30.110	1	**<0.001**	1.019	1.012	1.026

### 3.3. Statistical assumptions of logistic regression

The Box-Tidwell transformation was used to demonstrate linear relationships between the outcome Y-logit (Intention to leave) and each of the WRQoL and CBI predictors [49]. In addition, the variance inflation factors for the six WRQoL subscales ranged from Stress at Work (VIF = 1.5) to Job-Career Satisfaction (VIF = 2.4). The burnout subscales produced higher values for Work-Related Burnout (VIF = 3.85) and Personal Burnout (VIF = 2.79) and Client-related Burnout was lower (VIF = 1.27). All these values were within the bounds of acceptability (VIF < 5) [50].

## 4. Discussion

The aim of this paper was to explore the factors impacting on nurses' intention to leave the profession as well as burnout and work-related quality of life measures. Our key findings highlight the factors that impact nurses' intention to leave, including younger age, being male, phase of study, lower job career satisfaction and lower general wellbeing at work, alongside higher scores in work-related and client-related burnout. These were all uniquely predictive of an increased likelihood of reporting intentions to leave. The country, work setting, and area of nursing practice were not found to be significant.

Age was identified as a significant factor in intention to leave, with younger nurses, particularly those aged 18–29 and 30–39, being four times more likely than those aged 60+ to report intention to leave the profession. Those aged between 40 and 59 were also more likely to report intentions to leave than those aged 60+ years, although it could be argued that the oldest age group are less likely to leave as they may be planning for retirement and not new work. However, NMC registration numbers highlight that 52.1% of those leaving the register had done so earlier than initially planned [5]. Younger age is reported as a predictive factor for nurses' intention to leave the profession elsewhere in the literature [3],[16],[31],[32] and emphasizes the need for retention and wellbeing at work strategies to address the needs of younger nurses. Newly qualified nurses have also been identified as an at-risk group for leaving the profession [33],[36] and, while in the UK, there is a system of preceptorship [51]–[52] for newly qualified staff, there is a need to ensure that wellbeing needs are met to decrease the risk of burnout and early exit from the profession [53].

Being male also increased the likelihood of expressing an intention to leave. The number of male respondents in our sample was small (*n* = 167, 9.6%) and may have risked bias. However, only 10.9% of UK nursing registrants are male [54]. There are mixed findings on the impact of gender in other research, with an Italian study also finding that males were more likely to express intentions to leave [14], while females elsewhere were more likely to leave [17].

Significant differences emerged across the phases of the study, with those responding in Phase 6 significantly less likely to report intentions to leave than those in Phases 2, 3 and 4. Explanations for this difference could include the timings of the survey. As stated earlier, we cover Phases 2–6, and Phases 2–4 include the period from November 2020 to February 2022, with the survey repeated at 6-month intervals. During these phases, the impact of the COVID-19 pandemic was very much in evidence, with tiered geographical restrictions implemented in the UK in November 2020 and restrictions on visiting care homes and hospitals and wearing of face coverings still in force until early 2022 [55]. The impact of the COVID-19 pandemic on wellbeing, work-related quality of life and burnout in the health and social care workforce has been well documented [15],[20],[26],[55]–[57], and subsequent intentions to leave [4],[16],[18],[58]. In February 2022, the last restrictions in the UK were removed, with policy shifting towards ‘living with COVID’ [59] and our findings show that after this date in Phases 5 and 6, nurses were less likely to report intentions to leave. However, it should be noted that the percentage reporting an intention to leave remained high at 40% and 37.6% in Phases 5 and 6, respectively. It has also been reported that in the two years up until September 2022, the leaver rate for NHS nurses in the UK increased from 9% to 11.5% [6] and it may be possible that the reduction in numbers intending to leave may be a result of increased numbers having already left. High numbers of nurses in the US were also reported to have left during the COVID-19 pandemic [60] and a systematic review of intentions to leave during the pandemic suggested that nurses were the occupational group most likely to leave their profession [61].

Analysis of the Work-Related Quality of Life (WRQoL) data revealed that two components of the scale were also found to be significant predictors of intentions to leave, i.e., job/career satisfaction (*p* < 0.001) and general wellbeing at work (*p* < 0.024). Using the cut-off scores identified by Eastman and van Laar [44], Stress at Work and General Wellbeing were both found to be at the lower levels of quality of working life across the phases (*M* = 4.37, *SD* = 1.88 and *M* = 19.14, *SD* = 4.76, respectively). Stress at Work, however, did not appear as a significant factor in the final model, this could be the result of its correlation with Work-related Burnout [*r*(1506) = −0.60, *p* < 0.001]. Job stress has been found to affect job satisfaction in previous studies [3],[7] and to negatively impact quality of working life [43], increasing the risk of burnout [62] and the likelihood of intention to leave [63]. The principal component analysis summarized in Table 7 supports the idea of conceptual overlap between feelings of stress, mental wellbeing and burnout and this was also evident in the moderate to strong correlation between the extracted components which were labelled WRQoL and Burnout (*r* = 0.57). However, given the demonstrated psychometric properties of both the WRQoL [38],[44] and the CBI [64] the research team have opted to statistically document their similarity rather than attempt to combine these previously validated measures when examining the unique predictors of intention to leave.

Other studies have found an association between job satisfaction and intention to leave [3],[7]. In Sweden, as well as work-related stress and job/career satisfaction, difficulties with the home-work interface were also found to be a significant factor as well as caring for patients with COVID-19 [16].

There has been much discussion of work conditions in the UK in recent times, particularly in relation to pay, which affects job satisfaction [40],[65] and staffing levels [5],[16],[41], and they may therefore be expected to impact intentions to leave. In the final model, lower job/career satisfaction was found to significantly increase intention to leave but was also found to be highly positively correlated with working conditions [*r*(1687) = 0.73, *p* < 0.001], which may explain its omission as a significant factor in the final model. In Phase 6, respondents were asked about their perception of whether there were safe levels of staffing in their workplace. As indicated earlier, a high percentage of nurses felt that staffing levels were not safe in their area of work and, of these nurses, 49.5% expressed an intention to leave as opposed to 20.8% of nurses who believed their area to have safe staffing levels [*χ^2^*(1) =14.95, *p* < 0.001]. An international study across six countries found that perceived adequacy of staffing levels was a consistently significant predictor of intention to leave [66]. The negative impact of perceived low staffing levels on job satisfaction and intention to leave is reported in other studies [14],[41],[67].

In this study, using the cut-off scores cited by Creedy et al. [45], mean scores across the phases revealed that both work-related and personal burnout were moderate, whereas client-related burnout was low. This demonstrates that direct work with patients was not the main source of burnout, suggesting other factors had a greater impact, with studies elsewhere having found lower client-related burnout scores than personal and work-related burnout scores [62],[68]–[69]. Once burnout was added to the regression model (see Table 7), the probability of intention to leave was associated with both work-related and client-related burnout. Given the moderate mean score for personal burnout across all phases, it is perhaps surprising that this did not appear significant in the final model. However, this is potentially explained by the high correlation between Personal and Work-related Burnout [*r*(1507) = 0.78, *p* < 0.001]. The impact of burnout on intention to leave has been reported from several countries, showing that, while a range of factors affected the different nations individually, burnout was a consistent predictor of intention to leave across all countries [17]. Other studies have explored factors associated with burnout levels, including the complexity of cases and poor working environment [19] and workload and work-life balance [28]–[29],[70] and the consequent reported intention to leave. There is a pressing need to address burnout in the nursing workforce as it has been shown to impact on the quality and safety of care received by patients [68],[70],[71]. A recent European study across six countries asked nurses what interventions they felt would best support their wellbeing; most (79%) felt that the most important factor would be increasing staffing levels [21].

Our results show that a range of factors impacted nurses' intention to leave over five phases from November 2020 to February 2023, with 47.9% expressing an intention to leave the profession across the study. This is an important finding as intention is a predictor of nurses actually leaving the profession [8]. The need to retain nurses is highlighted globally [1],[2] and in the UK [6],[52]. While there was a small increase in nurses registered with the NMC in 2023 [5], there is evidence of nurses leaving the profession earlier than planned [5],[65] as well as a 32.5% reduction in applications to nursing degrees over the last 3 years in the UK [72]. Effective and transformational leadership has been identified as important and can be associated with lower intent to leave [67],[73]. Such leadership could be harnessed to address the high rates of burnout and lower wellbeing through effective collaboration with human resources and occupational health departments to implement strategies and services that will support the workforce.

### 4.1. Limitation and strengths

It was evident that numbers responding to the survey declined over the period, and it is possible that with other NHS and research surveys on the impact of COVID-19 that survey fatigue set in [74],[75]. A further potential limitation is the use of convenience sampling using online recruitment via Twitter (now X) and Facebook; however, while it has been argued that this increases the risk of bias [76],[77], it provided an effective means to access large numbers of respondents which would have been practically difficult to achieve otherwise.

The strengths of this study include the multiple data collection phases which allowed the exploration of changes in intention to leave, WRQoL and burnout over a period of time that covered nurses working through the COVID-19 pandemic until shortly before the World Health Organisation declared COVID-19 to be no longer a global health emergency [78]. Collecting cross-sectional data at each phase of the study was deemed preferable to a strict longitudinal design. Although longitudinal designs are powerful and helpful in assessing changes within individuals over time, this design was considered sub-optimal given the pragmatic constraints on data collection during the COVID-19 pandemic, the risk of high attrition rates over six data collection points and the desire of the research team to maximize response rates by offering respondents complete anonymity.

## 5. Conclusion

A range of complex factors contribute towards nurses' stated intentions to leave their profession, and, due to the severe pressure on services that predates but was worsened by the COVID-19 pandemic, interventions must be developed in collaboration with human resources and occupational health colleagues that support nurses' wellbeing to address the high burnout rates evident here. There is also a need to address the varied factors impacting on job satisfaction, including having a manageable workload, safe staffing levels alongside a work environment that promotes retention through adequate reward, conditions and effective leadership. Future research must consider the effectiveness of interventions on nurses' wellbeing and burnout levels as well as the subsequent impact on patient quality of care and safety and retention in the profession as well as within organizations.

## Use of AI tools declaration

The authors declare they have not used Artificial Intelligence (AI) tools in the creation of this article.
